# Comparing Results from 2-D and 3-D Phenotyping Systems for Soybean Root System Architecture: A ‘Comparison of Apples and Oranges’?

**DOI:** 10.3390/plants13233369

**Published:** 2024-11-29

**Authors:** François Belzile, Waldiodio Seck, Prabhjot Sanghera, Liwen Han, Pierre Dutilleul

**Affiliations:** 1 Département de Phytologie, Faculté des Sciences de l’Agriculture et de l’Alimentation (FSAA), Université Laval, Québec, QC G1V 0A6, Canada; francois.belzile@fsaa.ulaval.ca (F.B.); seckwaldiodio@yahoo.fr (W.S.); 2 Institut de Biologie Intégrative et des Systèmes (IBIS), Université Laval, Québec, QC G1V 0A6, Canada; 3 Department of Plant Science, Faculty of Agricultural and Environmental Sciences, McGill University, Macdonald Campus, Sainte-Anne-de-Bellevue, QC H9X 3V9, Canada; prabhjot.sanghera@mail.mcgill.ca (P.S.); liwen.han@mcgill.ca (L.H.)

**Keywords:** plant phenotyping, root systems, soybean, imaging technologies, growing media, spatial dimensionality

## Abstract

Typically, root system architecture (RSA) is not visible, and realistically, high-throughput methods for RSA trait phenotyping should capture key features of developing root systems in solid substrates in 3D. In a published 2-D study using thin rhizoboxes, vermiculite as a growing medium, and photography for imaging, triplicates of 137 soybean cultivars were phenotyped for their RSA. In the transition to 3-D work using X-ray computed tomography (CT) scanning and mineral soil, two research questions are addressed: (1) how different is the soybean RSA characterization between the two phenotyping systems; and (2) is a direct comparison of the results reliable? Prior to a full-scale study in 3D, we grew, in pots filled with sand, triplicates of the Casino and OAC Woodstock cultivars that had shown the most contrasting RSAs in the 2-D study, and CT scanned them at the V1 vegetative stage of development of the shoots. Differences between soybean cultivars in RSA traits, such as total root length and fractal dimension (FD), observed in 2D, can change in 3D. In particular, in 2D, the mean FD values are 1.48 ± 0.16 (OAC Woodstock) vs. 1.31 ± 0.16 (Casino), whereas in 3D, they are 1.52 ± 0.14 (OAC Woodstock) vs. 1.24 ± 0.13 (Casino), indicating variations in RSA complexity.

## 1. Introduction

In most major crops, including soybean, breeding for increased tolerance to abiotic stresses, many of which are exacerbated by climate change, has become a critical mission. A key to overcoming abiotic stresses lies in the development of cultivars with improved root systems, but the main impediment to this development is that roots are not readily visible [1,2]. Therefore, root system architecture (RSA) and its phenotyping are receiving greater attention, especially by the use of non-destructive procedures [3]. Some optical phenotyping systems using photography resort to transparent growth substrates (e.g., water, gel), which do not reproduce the physical constraints (e.g., compaction, resistance to penetration) of a solid substrate such as soil, thus leading to the exploration of alternative substrates [4]. In general, inexpensive and high-throughput phenotyping systems provide 2-D images of root systems. The non-destructive phenotyping of roots in 3D in the soil is possible with technologies such as X-ray computed tomography (CT) scanning [5,6,7], magnetic resonance imaging [8], and positron emission tomography [9], originally designed for diagnostic purposes in medicine. These technologies, provided that an appropriate analysis of images for plant roots is available, allow capturing a 3-D view of the RSA in situ non-invasively. In particular, the application of CT scanning for phenotyping a large number of plants has recently become more affordable, but the transition from 2-D to 3-D work opens the door to multiple questions and motivates the revisit of basic experimental guidelines.

Photography was used in [10] to apply a mostly non-destructive 2-D imaging approach to characterize the RSA for soybean plants at the V1 stage of development of the shoot and grown in a 1.5 cm layer of vermiculite trapped between two acrylic plates, called “rhizobox”. Using three plants per cultivar for 137 soybean cultivars, they measured numerous RSA traits, including total root length, and performed a genome-wide association study that identified candidate genes for RSA-related traits. As a preliminary 3-D follow-up, we grew in pots containing non-sieved sand the two soybean cultivars found to have the most contrasting RSAs in [10], namely Casino and OAC Woodstock. We used CT scanning technology to perform 3-D imaging of the root systems at a similar stage of development of the shoots, measured the total root length, and estimated the fractal dimension (FD) from the 3-D images reconstructed from plant–soil CT scanning data for each of the new experimental root systems. Our objective is to identify, given the differences between the experimental conditions, the results that are comparable (i.e., for which a comparison makes sense) between the two soybean RSA phenotyping systems. In other words, whereas plant scientists generally want to minimize, ideally eliminate, cultivation differences to make results from different studies comparable, we follow a different approach. Therefore, we also estimated FDs from 2-D photographs of soybean root systems in [10]. Eventually, we determine to which degree differences between soybean cultivars captured with the 2-D system are consistent with those observed in the more sophisticated—and likely more comprehensive—3-D system, and propose explanations.

## 2. Materials and Methods

### 2.1. Plant Material

We chose the cultivars Casino and OAC Woodstock for 3-D imaging of the soybean RSA because they were found to offer the strongest contrast in total root length when grown in rhizoboxes in 2D, with mean total root lengths (*n* = 3) of 21.2 cm for Casino and of 530.4 cm for OAC Woodstock [10]. This is a feature that we wanted to re-assess with triplicates in 3D using CT scanning technology for imaging, and therefore grew in pots three plants of each of these two soybean cultivars until they reached a stage of development of the shoots, similar to the study in 2D [10].

### 2.2. Two-Dimensional System

In view of the Supplementary Figures 1–4 in [10], the Authors describe their 2-D phenotyping system as follows, starting with the growing container. The rhizobox (40.6 H × 25.4 L × 1.5 W cm^3^) enclosed two separate acrylicic plates, which were covered with paper to create a shadowed condition for roots. Between the plates, a growing medium (i.e., vermiculite) was available. Thus, watering and fertilizer application could take place, plants developed, the development of root systems could be observed, and clean roots were accessible for analysis of the 2-D RSA. That was after soybean seeds were germinated on vermiculite in a Petri dish and a single germinated soybean seed was positioned at the top of a 1 cm layer of vermiculite in each rhizobox. After 10 days of growth, a time corresponding to vegetative stage V1 (first trifoliolate—one set of unfolded trifoliolate leaves), the top sheet of acrylic was removed, and the root systems were photographed using a NIKON D3000 camera (Nikon, Tokyo, Japan). The automated identification of primary and secondary roots in the root system was performed with the Automatic Root Image Analysis software [11]. The main experimental information about the 2-D soybean RSA phenotyping system, which is tentatively compared in this Brief Report with a 3-D soybean RSA phenotyping system (see below), is summarized in Table 1.

### 2.3. Three-Dimensional System

The RSA of the Casino and OAC Woodstock soybean cultivars was studied in the following 3-D phenotyping system. Three soybean plants per cultivar were grown in standard greenhouse conditions, in plastic pots (15 cm in top diameter and 13 cm in height), filled with a mineral soil (i.e., non-sieved sand). Seeds were sown, one per pot, close to the center, at about 3 cm in depth, and germinated directly in the pots in the new experiment. When the soybean seedlings reached vegetative stage V1, each plastic pot was CT scanned for its content (sand, roots) at the CT Scanning Laboratory for agricultural and environmental research on the Macdonald Campus of McGill University. The CT scanner is a Canon CT Aquilion Prime SP (Canon, Tokyo, Japan). The X-ray tube voltage and current were set at 135 kV and 150 mA, respectively; the physical size of a voxel is 0.21 × 0.21 × 0.2 mm^3^; and the volume CT scanned per pot is 1.156 dm^3^, distributed over 500 CT images with 512 × 512 pixels each. MATLAB R2023b codes (MathWorks, Natick, MA, USA) and ImageJ Fiji 1.53t software (National Institutes of Health, Bethesda, MD, USA) were used to process and analyze CT images, as in [7]. In addition, two videos, one video per cultivar, were produced in MATLAB R2023b from skeletal 3-D images of the root systems, and are made available as a supplement to the graphical results presented in Section 3.

### 2.4. Fractal Analyses

Free of the constraint of taking only integer values (0: point, 1: straight line, 2: plane, 3: volume) like Euclidean dimensions, FD provides a useful measure of structural complexity for many branching patterns. For the root system of a plant, the estimation of FD requires an image of the “skeleton (i.e., the root system reduced to a thickness of one unit). Depending on the framework (2-D vs. 3-D) and the technology involved, the original image type is a photograph or a CT image, so that a 2-D image of the skeletonized root system is used in the box-counting procedure or a skeletal 3-D image reconstructed from CT images is used in the cube-counting procedure for FD estimation [12]. Estimates of FD cannot exceed the values of 2.0 in the 2-D procedure and 3.0 in the 3-D procedure. In both cases, the higher the FD estimate, the more complex the structure of the root system. After skeletonizing the root systems shown in photographs in Supplementary Figure 4 in [10], we applied the FD estimation procedure in 2D [13]. Using skeletal 3-D images of root systems as reconstructed from CT scanning data, we applied the FD estimation procedure in 3D [7]. That provided a total of 12 FD estimates (i.e., three per cultivar per phenotyping system).

## 3. Results

**Figure 1:**
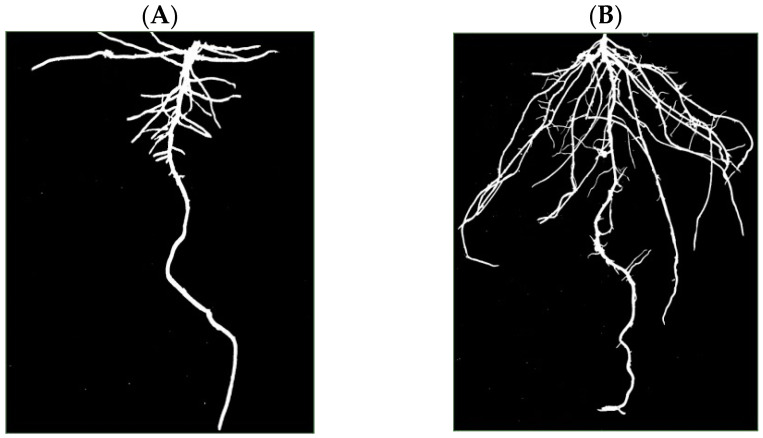
Characterization of soybean root system architecture in 2D, using rhizoboxes filled with vermiculite as a growing medium. Photograph of the root system for a soybean plant at the V1 stage of development of the shoot, from the cultivars (**A**) Casino and (**B**) OAC Woodstock (edited from Supplementary Figure 4 in [10]). V1: first trifoliolate—one set of unfolded trifoliolate leaves. The scale in panel (**B**) is about 5-fold larger than in panel (**A**).

**Figure 2:**
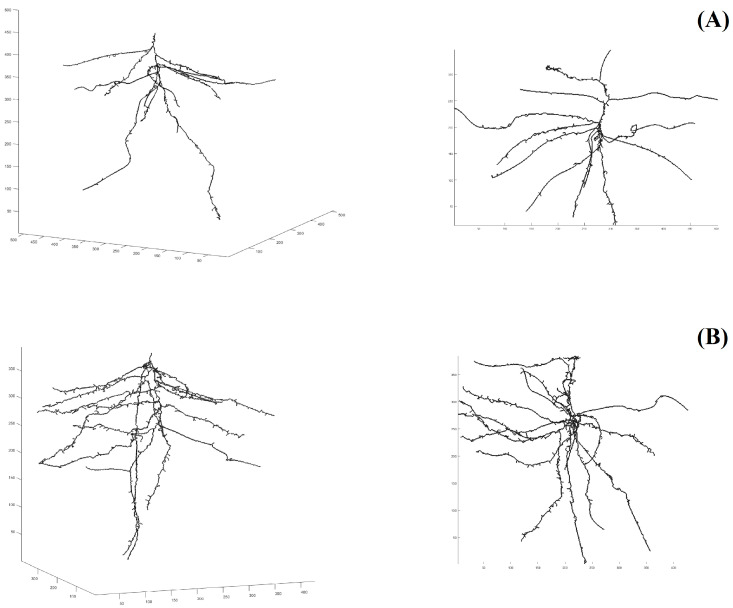
Characterization of soybean root system architecture in 3D, using pots filled with non-sieved sand as a growing medium: left, the lateral view and right, the top view of the skeletal 3-D image of the root system as reconstructed from plant–soil CT scanning data for one soybean plant at the V1 stage of development of the shoot, from the cultivars (**A**) Casino and (**B**) OAC Woodstock. V1: first trifoliolate—one set of unfolded trifoliolate leaves; CT scanned volume: 1.156 dm^3^; dimensions of a voxel: 0.21 (X) × 0.21 (Y) × 0.2 (Z) mm^3^.

In the 2-D phenotyping system, the soybean cultivars Casino and OAC Woodstock offered a stark, yet reproducible contrast in RSA ([10], Supplementary Figure 4); see Figure 1 here for an illustrative example with one plant per cultivar. The photograph of the Casino root system (Figure 1, left panel) shows a highly dominant primary root, few and relatively short secondary roots, and a very limited number of tertiary roots. The photograph of the OAC Woodstock root system (Figure 1, right panel) displays more branching and greater development, with a larger number of longer secondary roots, as well as tertiary roots. In the 3-D phenotyping system, the root system of OAC Woodstock appears more branched than the root system of Casino, in the lateral view (Figure 2, left panels) and the top view (Figure 2, right panels). For differences in actual 3-D space occupancy between the representatives of each of the two cultivars, the two videos in the Supplementary Material are available for watching, and confirm the observations made on lateral and top views in Figure 2.

From the 2-D and 3-D images, the two RSA traits of particular interest here, namely total root length and fractal dimension, were measured and estimated in the corresponding phenotyping systems. In the 2-D phenotyping system, the OAC Woodstock soybean seedlings developed root systems with a total root length 25 times longer on average than the Casino ones, i.e., 530.4 cm vs. 21.2 cm; see Supplementary Table 1 in [10]. Part of this marked difference in total root length is readily perceivable at the level of secondary roots, but another contribution comes from tertiary roots that are almost totally absent in cultivar Casino at this stage of development (Figure 1, left panel). The 3-D phenotyping system produced a clear difference in total root length between the two soybean cultivars, but the contrast is less marked: 152 ± 8 cm for OAC Woodstock vs. 88 ± 3 cm for Casino, than in the 2-D system.

Comparing FD estimates obtained from 2-D and 3-D images is not easy because the range of FD values in 2D is [0, 2], whereas it is [0, 3] in 3D. Nevertheless, the root systems of soybean cultivar OAC Woodstock show a greater structural complexity on average than those of Casino in both phenotyping systems: in 2D, 1.48 ± 0.16 (OAC Woodstock) vs. 1.31 ± 0.16 (Casino); in 3D, 1.52 ± 0.14 (OAC Woodstock) vs. 1.24 ± 0.13 (Casino). Thus, at an early stage of development, the Casino RSA tends to be closer to a straight line in 3D than in 2D, whereas space occupancy by a Woodstock root system, fragmented in 3D in the pot, falls halfway between those of a straight line and a plane.

## 4. Discussion

The results that we presented show that the size of differences in two key RSA traits (total root length, fractal dimension) between soybean cultivars Casino and OAC Woodstock depends on the phenotyping system, starting with a different growing medium: vermiculite in rhizoboxes in the 2-D system vs. a mineral soil (sand) in pots in the 3-D system. Sand opposes a greater resistance to developing roots, thereby decreasing their growth rate, whereas a lighter substrate, such as vermiculite, allows roots to grow faster. Pots also provide a less constraining spatial domain than rhizoboxes for the development of root systems in the first weeks after seed germination, allowing for a volumetric growth (downwards and in all directions laterally) vs. an almost planar growth.

Photography and CT scanning have their respective advantages and drawbacks in terms of imaging. The former reproduces and amplifies all that is visible to the naked eye, whereas the latter reveals the interior of the pot non-destructively and non-invasively. Roots can be missed in both cases. For high-throughput analysis of RSA traits, phenotyping systems need to be affordable and rapid, but the observed differences between cultivars must be real and meaningful, and not artifacts caused by the framework. By definition, phenotypic plasticity includes a genotype-by-environment interaction [14]. Thus, it is important to be conscious of the experimental conditions in which each imaging technology is used and the relevance of each type of measurement. Sophisticated imaging that would reflect the development of root systems in solid substrates in 3-D containers would trustfully pave the way for a field trial.

In a pilot study, we used CT scanning to characterize the RSA of soybean seedlings in a 3-D phenotyping system and found that the technology and subsequent graphical and quantitative analyses in 3D are capable of capturing key features of the RSA. By comparison with the results of the 2-D phenotyping system, the differences found between two contrasting cultivars in the main topology of the root system (i.e., predominance of the taproot, abundance of secondary roots, total root length, structural complexity) may be more authentic because of the dimensionality of the spatial domain and the nature of the growing medium. Ideally, it would therefore be preferable to use CT scanning-based 3-D root imaging in solid substrates in the future, but a number of factors including those below must be considered.

With modern CT scanners, the reconstruction time to obtain a stack of CT images from raw projection images has decreased tremendously, to less than 1 min for hundreds of CT images. At least two important challenges, however, remain: (i) isolating (segmenting) the root system from the growing medium in the stack of CT images (3-D array of CT scanning data); and (ii) matching the best soil (type: organic or mineral, conditions: wet or dry, sieved; yes or no) for root CT scanning, with the best soil for the development of the plant [12,15]. For example, at the end of a comprehensive study, calcined clay was found to be the best soil for the development of a high-throughput approach to characterizing rice root systems [16].

The results that we presented and discussed for soybean justify a clear and strong reminder to plant breeders: selection based on RSA traits should be made with due knowledge of the experimental conditions in which the phenotyping results are obtained (including the devices used for root imaging), and phenotyping results in an experiment may not be extrapolated without risk to other experimental conditions. In the perspective of an experiment with a large number of soybean cultivars (including ‘intermediates’ in addition to ‘extremes’ like Casino and OAC Woodstock), our results suggest that the rankings of cultivars based on total root length and fractal dimension would depend on the phenotyping system and differences in 3D may be smaller or larger than in 2D [10], depending on the soybean cultivars considered. This prediction is likely to hold for other crops (e.g., corn, pea, potato).

In closing, when referring to a published RSA phenotyping study in the literature and citing reported results in support of our own results, we should pay attention not only to the crop(s), cultivar(s), and trait(s) studied, but also to the experimental procedures and protocols that were applied and may impose constraints on the development of root systems and make phenotypic plasticity express itself over the studies. Otherwise, we would not necessarily compare ‘apples’ and ‘apples’, even though the cultivars are the same and the RSA traits studied are the same…

## Figures and Tables

**Table 1:** Main experimental information about the 2-D and 3-D soybean RSA phenotyping systems tentatively compared for their results (RSA: root system architecture).

	2-D System [10]	3-D System (SÈVE Project #299498 by Belzile et al.)
Cultivars	Casino, OAC Woodstock	Casino, OAC Woodstock
Number of plants per cultivar	3	3
Growing medium	Vermiculite	Non-sieved sand
Growing container	Rhizobox	Pot
	(40.6 H × 25.4 L × 1.5 W cm^3^)	(15 cm top diameter, 13 cm height)
Space constraints	Strong(er)	Weak(er)
Spatial dimension	Essentially 2D	3D (within the limits of a pot)
Seed germination	In Petri dishes	In pots
Followed by transplantation	Yes	No
Watering regime	Every other day	Every other day
Fertilizer application	Yes	No
Time of phenotyping	Vegetative stage V1	Vegetative stage V1
Imaging technology	Photography	CT scanning
Destructive procedure	Essentially (*) No	No
RSA traits	Total root length	Total root length
	Fractal dimension (estimated from photos—by Belzile et al.)	Fractal dimension (estimated from 3-D skeletal images)

V1: first trifoliolate—one set of unfolded trifoliolate leaves; CT: computed tomography. (*) Except if roots attached to the acrylic sheet detached for photography break while the acrylic sheet is being detached.

## Data Availability

The soybean genomic data are publicly available at https://figshare.com/s/2eb426e0bb988b19e6ca and https://doi.org/10.6084/m9.figshare.12982886.

## Supplementary Materials

The following supporting information can be downloaded at https://www.mdpi.com/article/10.3390/plants13233369/s1. Two videos (.AVI files), one per soybean cultivar, were produced in MATLAB (MathWorks, Natick, MA, USA) from skeletal 3-D images of the root systems, and are made available as a supplement to the graphical results presented for the 3-D phenotyping system in Figure 2 in the manuscript. Video S1: Casino cultivar. Video S2: OAC Woodstock cultivar.
